# Biomimetic Superhydrophobic Surfaces: From Nature to Application

**DOI:** 10.3390/ma18122772

**Published:** 2025-06-12

**Authors:** Yingke Wang, Jiashun Li, Haoran Song, Fenxiang Wang, Xuan Su, Donghe Zhang, Jie Xu

**Affiliations:** 1 Key Laboratory of Micro-Systems and Micro-Structures Manufacturing of Ministry of Education, Harbin Institute of Technology, Harbin 150080, China; wangyingke66@hit.edu.cn (Y.W.); 24s109373@stu.hit.edu.cn (J.L.); 24b909113@stu.hit.edu.cn (H.S.); 24b909114@stu.hit.edu.cn (F.W.); 2 Zhengzhou Research Institute, Harbin Institute of Technology, Zhengzhou 450008, China; suxuan@hit.edu.cn; 3 School of Mechatronics Engineering, Harbin Institute of Technology, Harbin 150001, China

**Keywords:** biomimetic, superhydrophobic, wettability, water-repellent, hierarchical structures

## Abstract

Research on bionic superhydrophobic surfaces draws inspiration from the microstructures and wetting mechanisms of natural organisms such as lotus leaves, water striders, and butterfly wings, offering innovative approaches for developing artificial functional surfaces. By synergistically combining micro/nano hierarchical structures with low surface energy chemical modifications, researchers have devised various fabrication strategies—including laser etching, sol-gel processes, electrochemical deposition, and molecular self-assembly—to achieve superhydrophobic surfaces characterized by contact angles exceeding 150° and sliding angles below 5°. These technologies have found widespread applications in self-cleaning architectural coatings, efficient oil–water separation membranes, anti-icing materials for aviation, and anti-biofouling medical devices. This article begins by examining natural organisms exhibiting superhydrophobic properties, elucidating the principles underlying their surface structures and the wetting states of droplets on solid surfaces. Subsequently, it categorizes and highlights key fabrication methods and application domains of superhydrophobic surfaces, providing an in-depth and comprehensive discussion.

## 1. Introduction

Through long-term evolution and natural selection, organisms in nature have developed intricate and sophisticated surface microstructures to adapt to harsh environments and enhance survival. These microstructures interact with liquids to create unique solid–liquid interfacial wetting properties [1,2,3,4]. Such special interfacial wettability endows organisms with diverse surface functionalities, including the self-cleaning ability of lotus leaves [5,6], anisotropic wettability of rice leaves [7,8], anti-fogging capability of mosquito eyes [9,10], and water-collecting function of desert beetles [11,12]. The remarkable wetting phenomena observed in nature have provided abundant inspiration and theoretical foundations for the design and fabrication of biomimetic functional surfaces by humans.

Over the past three decades, the rapid advancement of wettability research has led to the development of thousands of biomimetic surfaces exhibiting unique wetting properties [13,14]. Among these, superhydrophobic surfaces—characterized by water contact angles exceeding 150°—have garnered significant attention due to their exceptional water-repellent capabilities [15,16,17,18,19]. The understanding of superhydrophobic surfaces was initially inspired by the lotus leaf. In 1997, German botanists Wilhelm Barthlott and Christoph Neinhuis [20,21] investigated the microstructure and chemical composition of the lotus leaf surface, attributing its superhydrophobicity to the synergistic effect of micron-scale papillae and a low-surface-energy waxy layer. Subsequent advancements in electron microscopy enabled more detailed studies; notably, in 2002, Lei Jiang et al. [7] discovered that the lotus leaf’s papillae are adorned with numerous nanostructures. They concluded that the hierarchical micro/nanostructure, combined with the waxy layer, is responsible for the leaf’s high static contact angle (>150°) and low sliding angle (<10°). Further research has identified various organisms exhibiting superhydrophobic properties. For instance, the legs of water striders possess nanoscale grooves that trap air, forming a stable air cushion allowing them to walk on water surfaces [22,23]. Similarly, the dense micron-scale papillae on rose petals cause water droplets to partially penetrate the surface, resulting in a highly adhesive superhydrophobic state [24,25]. These findings underscore that constructing rough structures with low solid–liquid contact area fractions, coupled with low-surface-energy chemical modifications, is crucial for achieving superhydrophobicity [26,27,28].

The hydrophobicity of a solid surface is commonly quantified by the contact angle, which is the angle formed at the junction of the solid, liquid, and gas phases; it is also the equilibrium of surface tensions at the three-phase interface [29]. Based on the high repulsive properties of superhydrophobic surfaces to water droplets, the surfaces show a wide range of applications in the fields of low adhesion, self-cleaning, and anti-pollution [30,31,32]. For example, due to the difficulty of water droplets penetrating superhydrophobic surfaces, electronic devices can be kept dry, thereby preventing corrosion caused by moisture on the surface [33]. Thanks to their self-cleaning properties, superhydrophobic surfaces help maintain cleanliness on transparent materials like textiles and glass by preventing dust accumulation [34,35]. Additionally, their anti-fouling characteristics make them suitable for application on marine vessels, where they inhibit microbial adhesion and biofouling, thereby reducing corrosion from prolonged water exposure and effectively extending the service life of ships [36]. Inspired by the exceptional wetting properties observed on biological surfaces in nature, biomimetics has not only provided abundant inspiration for the structural design of superhydrophobic surfaces but also laid a theoretical foundation for their practical applications. Since the advent of the 21st century, advancements in nanotechnology and surface science have shifted the focus of superhydrophobic surface research from fundamental theory to application development, leading to significant breakthroughs in areas such as anti-icing and oil–water separation, as well as demonstrating vast potential for diverse industrial applications.

**Figure 1:**
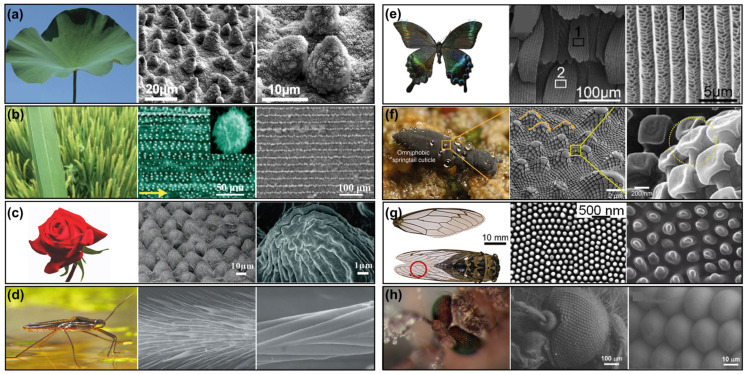
Photographs and microstructures of various living organisms exhibiting special wettability: (**a**) lotus leaf [1]; (**b**) rice leaf [7]; (**c**) rose petal [25]; (**d**) water skater [22,23]; (**e**) butterfly wing (black and white boxes marked with numbers 1 and 2 show the SEM images of wings) [37]; (**f**) springtails (yellow circle show the nanoarray on the micrometer structure of the epidermis) [38]; (**g**) cicada’s wings (red circle show the digital photo of cicada wings) [39]; (**h**) mosquito eyes [9].

This article systematically reviews the significant advancements in superhydrophobic surfaces. It begins by introducing natural organisms exhibiting exceptional superhydrophobic properties (Figure 1) and classical wetting models. Subsequently, it examines various fabrication methods for creating superhydrophobic surfaces. The article then focuses on the diverse practical applications of these surfaces. Finally, it briefly discusses the challenges faced and the future prospects in the field of superhydrophobic surfaces.

## 2. Design Strategy of Superhydrophobicity

### 2.1. Superhydrophobicity in Nature

Wettability refers to the interaction between a liquid and a solid surface, determined by both the surface’s chemical composition and its microstructure, and it is typically quantified by the contact angle. Over millions of years, organisms in nature have evolved sophisticated strategies to regulate wettability. For example, the lotus leaf achieves superhydrophobicity through the dual structure of micron-sized papillae and nanometer-sized wax crystals, and its self-cleaning effect allows water droplets to carry away surface pollutants [40]; the water strider walks on the surface of water by trapping an air layer through the oriented arrangement of bristles and nano-grooves on its legs, forming a strongly hydrophobic surface [22]; the periodic scale structure of a butterfly’s wings achieves superhydrophobicity and structural colors at the same time [37]; and the springtail shows rare superomniphobicity due to hexagonal pit arrays and nanoparticles on the body surface [38]. These organisms provide a template for the development of artificial superhydrophobic surfaces through the synergistic interaction of micro- and nanostructures with low surface energy materials.

#### 2.1.1. Lotus Leaf

The lotus leaf stands as a quintessential example of a superhydrophobic biological surface, with its remarkable water repellency arising from the synergistic interplay between its unique multiscale micro/nanostructures and a low-surface-energy wax coating (Figure 1a) [6]. On the microscale, the leaf surface is adorned with conical papillae, each measuring approximately 5–15 μm in diameter and 10–20 μm in height. These structures constitute the primary level of surface roughness. Overlaying these papillae are nanoscopic wax crystals, about 100–200 nm in size, forming the secondary level of fine structure. This hierarchical micro/nanostructure significantly enhances the surface roughness, resulting in an air fraction (fair) of 95–98% and a solid fraction (fs) of 2–5% on the surface of a lotus leaf. The interstitial spaces between papillae trap air, creating a continuous air cushion that underpins the Cassie–Baxter wetting state. Furthermore, the epicuticular wax layer predominantly comprises C29–C33 alkanes, with exposed methyl groups that reduce the surface energy to approximately 25 mN/m. This precise structural and chemical configuration results in a static water contact angle reaching up to 162° and a sliding angle of less than 5°, enabling water droplets to effortlessly roll off the surface, effectively removing contaminants. This phenomenon is famously known as the “lotus effect” [40].

#### 2.1.2. Rice Leaf

The superhydrophobicity of rice leaf surfaces originates from their distinctive anisotropic micro/nanostructured hierarchical architecture. Aligned along the leaf veins are parallel microgrooves spaced 50–100 μm apart and 20–30 μm deep. Each groove is overlaid with flake-like wax crystals measuring 30–50 nm in thickness (*f*_air_ ~ 92–95%, *f*_s_ ~ 5–8%). This oriented, multiscale structure imparts pronounced direction-dependent wetting behavior (Figure 1b) [7,8]. In the direction parallel to the veins, water droplets exhibit low sliding angles and high mobility, whereas in the perpendicular direction, they experience strong pinning effects. This anisotropy arises from the directional regulation of the three-phase contact line by the microgrooves. The parallel alignment of nanowax crystals further amplifies this effect, resulting in a contact angle difference of up to 20° between the two directions. Such intricate biological design not only facilitates rainwater to wash along predetermined paths but also guides the directed collection of dew, providing an ideal model for developing novel surfaces capable of directed liquid transport.

#### 2.1.3. Rose Petal

The rose petal surface exhibits unique high-adhesion superhydrophobic properties, with a static contact angle reaching up to 152° while demonstrating exceptionally high adhesion. This special wetting behavior originates from its ingeniously designed hierarchical structure. The rose petal surface is densely covered with hemispherical papillae spaced 10–20 μm apart, each featuring a flat plateau of 5–8 μm in diameter at its apex and adorned with radial nanofolds measuring 200–500 nm (*f*_air_ ~ 60–68%, *f*_s_ ~ 32–40%) (Figure 1c) [24,25]. This structure causes water droplets to adopt a Cassie-impregnating mixed wetting state: the tops of the papillae directly contact water molecules, forming a high-adhesion Wenzel state, while the inter-papillary spaces trap air, creating a superhydrophobic Cassie state. The flat plateaus increase the solid–liquid contact area, and the nanofolds generate multiple triple-phase line pinning effects, resulting in contact angle hysteresis exceeding 40°. This perfect synergy between structure and function not only achieves the “suspended droplet” superhydrophobic characteristic but also aids in capturing dew droplets on insect surfaces during pollination, demonstrating how biological evolution precisely adapts to specialized wettability requirements. It serves as an important bio-inspired prototype for developing novel liquid-manipulating interfaces.

#### 2.1.4. Water Skater

The superhydrophobicity of water strider legs is one of the most engineering-inspiring biological surfaces in nature, with their unique structural design enabling efficient walking and jumping on water (Figure 1d) [22,23]. This remarkable superhydrophobic property stems from the synergistic effect of hierarchical structures and specialized surface chemistry. Each water strider leg is densely covered with approximately 20,000 bristles per square millimeter, each measuring 3–5 μm in diameter. These bristles are further decorated with spiral nanogrooves spaced 200–300 nm apart, forming a micro/nano dual-scale roughness (*f*_air_ ~ 99.8–99.95%, *f*_s_ ~ 0.05–0.2%). This intricate architecture reduces the actual contact area between the leg surface and water to less than 0.5%. Meanwhile, the 20–30° inclined arrangement of the bristles and the periodic undulations of the nanogrooves collectively generate strong capillary forces, locking water droplets in a Cassie-impregnating state, where water molecules partially infiltrate the gaps between bristles without penetrating to the base. The surface is coated with hydrocarbon wax, which, combined with the structural enhancement, allows a single leg to support up to 15 times the insect’s body weight (withstanding pressures of 1.2 kPa). This perfect synergy between structure and chemistry not only grants the water strider extraordinary aquatic mobility (reaching speeds of 1.5 m/s) but also ensures exceptional dynamic stability. It serves as an ideal design blueprint for applications such as bio-inspired aquatic robots.

#### 2.1.5. Butterfly Wing

Butterfly wings represent a classic biological surface in nature that combines superhydrophobicity with structural coloration. Their unique micro/nano structures, optimized through long-term evolution, exhibit exceptional water-repellent properties and optical characteristics (Figure 1e) [37,41]. The superhydrophobicity of butterfly wings stems from the synergistic effect of their intricate hierarchical micro/nano structures and hydrophobic wax layers. The surface is covered with regularly arranged scales (50–100 μm in size), each featuring parallel, ridge-like structures spaced 1.5–2 μm apart. These primary ridges further branch into nanoscale lamellae with a thickness of 50–100 nm, forming a network of 100–300 nm cavities (*f*_air_ ~ 95–98%, *f*_s_ ~ 2–5%). This hierarchical architecture results in an extremely low solid–liquid contact area fraction. Coupled with the low-surface-energy wax layer, it stabilizes the Cassie–Baxter wetting state. The distinctive ridge–cavity design not only generates structural coloration but also endows the surface with outstanding dynamic performance. Such a biologically inspired dual-functional integration of optical and hydrophobic properties provides an ideal model for developing novel intelligent surfaces.

#### 2.1.6. Springtails

The springtail’s cuticle exhibits an exceptionally rare omniphobic property in nature (water contact angle 165°, oil contact angle > 150°), with its unique performance stemming from an ingeniously designed hierarchical composite structure (Figure 1f) [38,42]. The springtail’s surface is densely covered with hexagonal dimple arrays (0.5–2 μm in diameter), each embedded with 50–100 nm hydrophobic protein–chitin composite nanoparticles that further feature 10–20 nm secondary protrusions (*f*_air_ ~ 98.5–99.2%, *f*_s_ ~ 0.8–1.5%). This micro/nano hierarchical architecture drastically reduces the solid–liquid contact area. Particularly noteworthy are the 20–30 nm cantilever structures at the dimple edges, which mechanically pin the solid–liquid–gas triple-phase contact line, endowing the surface with remarkable anti-adhesion capabilities. It can repel highly viscous soil mucus (>100 cP) and 5–10 μm fungal spores while maintaining superhydrophobicity even after 1000 cycles of sand abrasion. This biological design—simultaneously water-repellent, oil-repellent, and anti-adhesive—provides a biomimetic template for developing coatings suitable for extreme environments (e.g., crude oil contamination, high-humidity soil). Its core innovation lies in achieving perfect unification of super-repellency and mechanical stability through nano-cantilever structures.

#### 2.1.7. Cicada’s Wings

The cicada wing achieves remarkable superhydrophobicity (contact angle 162°, sliding angle < 3°) through the synergistic effect of precisely arranged nanopillar arrays (diameter: 80–120 nm, height: 200–300 nm, spacing: 150–200 nm) and an ultrathin wax layer (*f*_air_ ~ 87–90%, *f*_s_ ~ 10–13%) (Figure 1g) [39,43]. The hexagonal, close-packed nanostructure reduces the solid–liquid contact area to below 7%, while its unique gradient design ensures over 95% visible light transmittance and exceptional dynamic water-repellent capability. Notably, the nanopillars’ aspect ratios and hemispherical apexes perfectly balance mechanical strength and hydrophobicity, enabling the cicada wing, with an areal density of merely 0.01 g/cm^3^, to maintain high self-cleaning efficiency and anti-bacterial adhesion performance. This biological design integrates optical transparency, ultralight weight, and superhydrophobicity into a single system, offering an ideal biomimetic prototype for next-generation applications like transparent aircraft coatings. Its breakthrough lies in achieving multifunctional synergy through nanoscale structural gradients—a feat challenging for conventional materials.

Superhydrophobic phenomena in nature (e.g., the “self-cleaning effect” of lotus leaves and the “water-walking” ability of water striders) provide key design blueprints for the fabrication of artificial superhydrophobic surfaces through their delicate micro- and nano-hierarchical structures and low-surface-energy chemical compositions. These biological prototypes (e.g., the composite structure of micron papillae and wax crystals in the lotus leaf, and the array of oriented bristles in the legs of water striders) reveal a synergistic mechanism of ”structural roughening + surface chemical modification”, which has directly inspired the development of technical pathways for the construction of biomimetic micro- and nano-morphologies through laser etching, the replication of porous networks by sol-gel methods, and the reduction in surface energy by the self-assembly of fluorosilane. For example, the use of templates to replicate the self-healing superhydrophobic surface of lotus leaf micro- and nano-papillary structures has achieved true biomimicry. This logic of “biological observation, mechanism analysis, artificial reproduction, and performance optimization” not only verifies the intelligent design of natural evolution, but it also promotes the leap of superhydrophobic technology from the laboratory to the practical application in the fields of architecture, energy, and healthcare.

### 2.2. Wetting on Solid Surfaces

#### 2.2.1. Young’s Model

**Figure 2:**
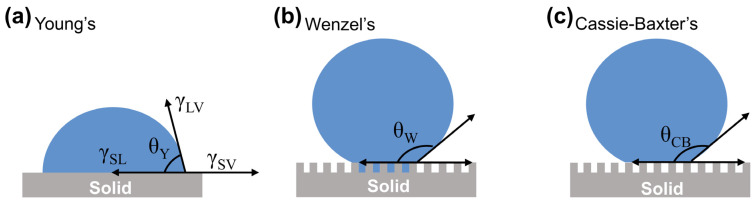
Contact model of liquid on solid surface: (**a**) Young’s model; (**b**) Wenzel model; (**c**) Cassie–Baxter model.

Young’s model (Figure 2a) reflects the wettability state of a droplet on an ideally smooth solid surface (flat and chemically homogeneous), where the contact angle depends mainly on factors such as the free energy of the solid surface and the interfacial tension. The model was proposed by Thomas Young in 1805 and established the famous Young’s equation [44], which laid the theoretical foundation for the study of solid surface wettability:

(1) $$\gamma_{sv}=\gamma_{sl}+\gamma_{lv}cos\theta$$

In the equation, *γ_sv_* represents the solid–gas interfacial tension, *γ_sl_* denotes the solid–liquid interfacial tension, *γ_lv_* stands for the liquid–gas interfacial tension, and *θ* is the intrinsic contact angle of a liquid droplet on an ideal solid surface. The magnitude of *θ* reflects the wettability of the solid surface. A surface with *θ* > 90° is typically defined as a lyophobic (liquid-repellent) surface, while a surface with *θ* < 90° is classified as a lyophilic (liquid-wetting) surface.

#### 2.2.2. Wenzel Model

However, when a droplet is in contact with a rough solid surface, the droplet fills the grooves of the rough structure, and the ideal state of Young’s equation cannot be applied to the model. Therefore, Wenzel introduced the roughness factor into the study of the wettability of solid surfaces in 1836 and established the famous Wenzel model (Figure 2b) with the equation shown in (2) [45]:

(2) $${cos\theta }^{*}=\frac{r(\gamma_{sv}-\gamma_{sl})}{\gamma_{lv}}=r\cos\theta$$

In the equation, θ* represents the apparent contact angle of the droplet on the rough surface, *θ* denotes the intrinsic contact angle on an ideal smooth solid surface, and *r* is the roughness factor, defined as the ratio of the actual contact area to the projected area between the droplet and the solid surface. Since the actual area of a rough surface always exceeds its projected area, *r* > 1. The introduction of surface roughness amplifies the wettability of the solid surface. When *θ* < 90°, θ* < *θ*, indicating that hydrophilicity strengthens with increasing roughness. When *θ* > 90°, θ* > *θ*, demonstrating that hydrophobicity enhances with greater roughness.

#### 2.2.3. Cassie–Baxter Model

Young’s model (smooth surfaces) and Wenzel’s model (homogeneous roughness) assume equilibrium states with full liquid contact, failing to explain metastable Cassie states in natural superhydrophobic surfaces (e.g., lotus leaves).

In 1944, Cassie and Baxter proposed a new theory of wettability based on Wenzel’s model, in which they argued that the contact between a droplet and a solid surface is not limited to a single solid–liquid interface, but that a large air layer exists in the grooves of the solid structure underneath the droplet, forming a composite interface with solid–liquid–gas three-phase contact (Figure 2c), and established the famous Cassie–Baxter Equation (3) [46]. The model successfully explains the phenomenon of water droplets spontaneously rolling off the surface of a lotus leaf.

cosθ* = *f*_1_cos*θ*_1_ + *f*_2_cos*θ*_2_ (3)

In the equation, θ* represents the apparent contact angle on the rough solid surface, *θ*_1_ and *θ*_2_ denote the intrinsic contact angles of the droplet on an ideal solid surface and in air, respectively, and *f*_1_ and *f*_2_ are the area fractions of solid–liquid and air–liquid interfaces, satisfying *f*_1_ + *f*_2_ = 1. Since the intrinsic contact angle of the droplet in air (*θ*_2_) is 180°, the Cassie–Baxter equation can be simplified to:

cosθ* = *f*_1_(cos*θ*_1_ + 1) − 1 (4)

This theory proposes that liquid droplets cannot penetrate the air layers trapped within the microstructures of the solid surface, resulting in a composite solid–liquid–gas triple-phase contact interface. It successfully explains non-wetting superhydrophobic phenomena. In the equation, *f*_1_ is related to the surface roughness of the solid substrate. As roughness increases, *f*_1_ decreases, leading to an increase in the apparent contact angle (θ*) of the droplet on the solid surface.

Thus, enhancing surface roughness can effectively improve liquid repellency. Furthermore, the equation demonstrates that the wettability of a solid surface depends on both its microscopic topography and surface chemical composition.

However, the Cassie–Baxter model requires a uniform distribution of surface chemical composition and morphology, whereas the coexistence of micro- and nanoscale features in actual multilevel structures leads to differences in local wettability, and even random roughness makes it impossible to accurately define the solid–gas area fraction. In addition, the Cassie–Baxter model assumes a uniform distribution of contact line energy, but it is observed that the sub-stable Cassie–Baxter state is prone to transition to the Wenzel state. Therefore, for irregular surfaces, a combination of hysteresis and sliding angles is required for a complete characterization.

#### 2.2.4. Contact Angle Hysteresis and Sliding Angle

In addition to static contact angle measurements, dynamic contact angles serve as crucial criteria for evaluating solid surface wettability, and they are typically characterized by contact angle hysteresis and sliding angle [47,48].

**Figure 3:**
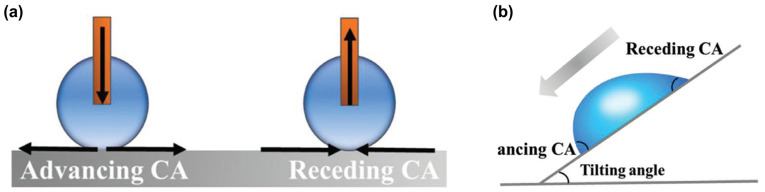
(**a**) Schematic diagram of the advancing angle and receding angle of a droplet on a solid surface; (**b**) Schematic diagram of the tilting angle of a droplet on an inclined solid surface [47].

Contact angle hysteresis (Δ*θ*) is typically defined as the difference between the advancing contact angle and receding contact angle of a droplet on a solid surface (Figure 3a) [49,50].

The advancing contact angle is measured by the increasing droplet volume, during which the three-phase contact line expands outward. At this stage, the solid–liquid interface replaces the solid–gas interface, and the resulting angle is termed the advancing contact angle (*θ_Adv_*). The receding contact angle is measured by the decreasing droplet volume, causing the contact line to retract inward. Here, the solid–gas interface replaces the solid–liquid interface, forming the receding contact angle (*θ_Rec_*). The magnitude of contact angle hysteresis reflects the droplet’s ability to detach from the surface. A small Δ*θ* indicates that the liquid droplets are easier to detach from the solid surface. Conversely, the larger Δ*θ* is, the more difficult it is for the liquid droplet to separate from the solid surface [51,52].

The sliding angle (SA) is another critical parameter for characterizing the dynamic wetting behavior of droplets on solid surfaces (Figure 3b). It is defined as the minimum tilt angle between the solid surface and the horizontal plane at which a deposited droplet begins to roll off. SA decreases with reduced contact angle hysteresis (Δ*θ*) [47,53]. In 1962, Furmidge [54] established the theoretical framework for predicting droplet mobility by correlating SA with surface properties.

(5) $$\mathrm{mg}(\sin\alpha )/w=\gamma_{lv}\left({cos\theta }_{Rec}-{cos\theta }_{Adv}\right)$$

In the equation, *m* represents the droplet mass, *g* denotes gravitational acceleration, *w* is the droplet perimeter, *θ_Adv_* and *θ_Rec_* are the advancing and receding contact angles, respectively, indicating the liquid–vapor surface tension, and *α* is the sliding angle of the droplet on the solid surface. A smaller sliding angle indicates better liquid repellency of the surface, facilitating easier droplet roll-off.

## 3. Progress in Preparation Methods of Superhydrophobicity

Based on the aforementioned design strategies, constructing micro/nano rough structures and applying low-surface-energy chemical modifications are fundamental to achieving superhydrophobicity. The micro/nano roughness enhances surface roughness and reduces the solid–liquid contact area, while the low-surface-energy treatment decreases the solid’s surface free energy, thereby improving hydrophobicity. After two decades of extensive research, numerous methods for fabricating superhydrophobic surfaces have been developed. Based on their working principles and process characteristics, these methods can be classified into three main categories: (1) Physical methods utilize physical energy (e.g., light, plasma) to directly construct rough structures; (2) chemical methods employ chemical reactions (e.g., redox, hydrolysis–condensation) to generate surface roughness; and (3) self-assembly techniques rely on weak intermolecular interactions to form thermodynamically stable, ordered structures. A detailed classification is outlined below.

### 3.1. Physical Methods

#### 3.1.1. Lithography

**Figure 4:**
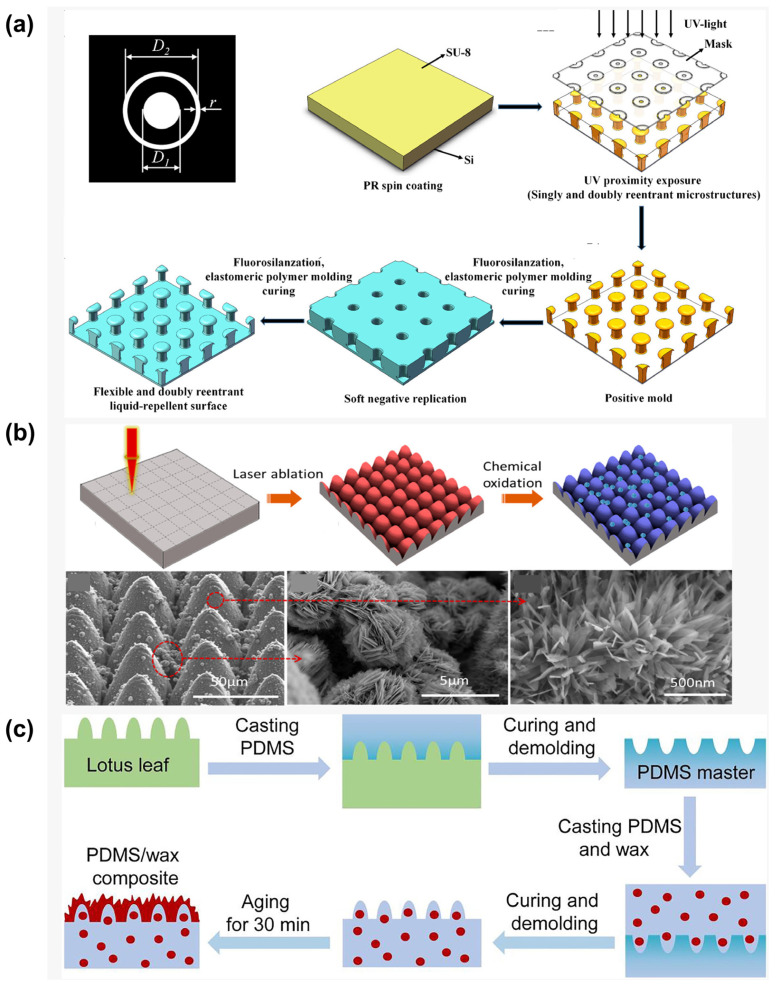
Preparation of superhydrophobic surfaces by physical methods: (**a**) lithography [55]; (**b**) laser Etching [60]; (**c**) template method [40].

Photolithography is a high-precision technique for creating superhydrophobic surfaces, employing photoresist patterning and subsequent transfer processes to construct micro/nano hierarchical structures (Figure 4a) [55]. The procedure involves spin-coating a photoresist layer onto the substrate, selective UV/EUV exposure through a photomask, developing it to reveal micrometer-scale patterns, using plasma etching to transfer the patterns to the substrate while generating nanoscale roughness, and then using fluorosilane modification to reduce surface energy. This method offers exceptional morphological control and high reproducibility.

Through photolithography, Fan et al. [56] demonstrated a simple, effective strategy to fabricate superhydrophobic surfaces on silicon wafers, exhibiting low contact time for impacting droplets. The surface featured micropillar arrays (10 μm diameter, 45 μm height) modified with liquid-like perfluoropolyether (PFPE) chains possessing high molecular flexibility. The combination of high-aspect-ratio micropillars and low solid fraction endowed the surface with superior superhydrophobicity. Furthermore, the liquid-like coating’s low contact angle hysteresis and enhanced interfacial slip significantly improved the surface’s dynamic repellency performance. Building on photolithographic techniques, Wang et al. [57] developed robust liquid-repellent surfaces with springtail-inspired nanoscale doubly reentrant structures using a simplified colloidal lithography process. The nanoscale features ensured sufficient triple-phase contact line density at a minimal solid–liquid contact fraction, thereby reinforcing capillary forces for liquid suspension. The synergistic integration with doubly reentrant topography maximized the upward component of capillary forces. The exceptionally stable solid–liquid–air composite interface enabled the nanoscale doubly reentrant surface to maintain outstanding repellency against high-speed droplet impacts, including water droplets at Weber number 257 and ethylene glycol droplets at Weber number 306.

#### 3.1.2. Laser Etching

Compared to photolithography, laser ablation represents a more efficient and versatile fabrication approach. This technique utilizes high-energy laser beams to create periodic micro/nano hierarchical structures on various substrates, including metals, polymers, and ceramics. Its unique, non-thermal melting characteristics prevent material thermal damage (Figure 4b) [58]. By precisely controlling laser parameters such as scanning speed, pulse overlap rate, and defocusing amount, researchers can accurately tune structural morphology parameters, which, when combined with fluorosilane modification, yield superhydrophobic surfaces with high contact angles.

Yang et al. [59] employed an integrated approach combining nanosecond/femtosecond laser processing with silanization treatment to fabricate multilevel periodic micro/nano structures on aluminum substrates. Within the smallest grid units, convex ridges define the periphery while the central regions feature dense micro/nano structures created by femtosecond laser etching. The subsequent silanization process effectively reduces the surface free energy of laser-treated samples, resulting in aluminum surfaces demonstrating superior superhydrophobicity and corrosion resistance. Pan et al. [60] demonstrated another innovative approach, combining ultrafast laser ablation with chemical oxidation to produce tri-scale micro/nano superhydrophobic surfaces on copper substrates. This novel surface architecture consists of periodic microcone arrays composed of densely grown nanograss interspersed with distributed microflowers. The engineered surface exhibits exceptional Cassie-state stability with a critical Laplace pressure reaching 1450 Pa. It is a crucial performance metric for effective anti-icing capability.

#### 3.1.3. Template Method

The template method represents an efficient biomimetic strategy for fabricating superhydrophobic surfaces by precisely replicating the hierarchical micro/nanostructures of biological prototypes through physical transfer processes (Figure 4c) [40,61]. This approach involves three key steps: (1) casting a flexible material onto a biological template to create a negative mold, (2) secondary transfer printing to produce a positive surface featuring micrometer-scale papillae and nanometer-scale wrinkles, and (3) fluorosilane vapor deposition to reduce surface energy. This technique enables high-fidelity reproduction of biological structural characteristics with excellent batch-to-batch consistency, making it particularly suitable for large-area superhydrophobic functionalization of flexible substrates.

As shown in Figure 4c, Wang et al. [40] developed a self-healing superhydrophobic surface mimicking natural lotus leaves through a simple double-replication process inspired by the wax-secretion mechanism of plant leaves. By incorporating n-nonadecane into a microstructured polydimethylsiloxane (PDMS) matrix, they created a biomimetic surface that replicates the self-repairing function of natural systems. The combination of n-nonadecane’s relatively low melting point and the high chain mobility of PDMS enables the damaged surface to restore its superhydrophobicity through wax secretion from the matrix, demonstrating a self-healing mechanism analogous to that observed in natural plant leaves.

### 3.2. Chemical Methods

#### 3.2.1. Chemical Etching

**Figure 5:**
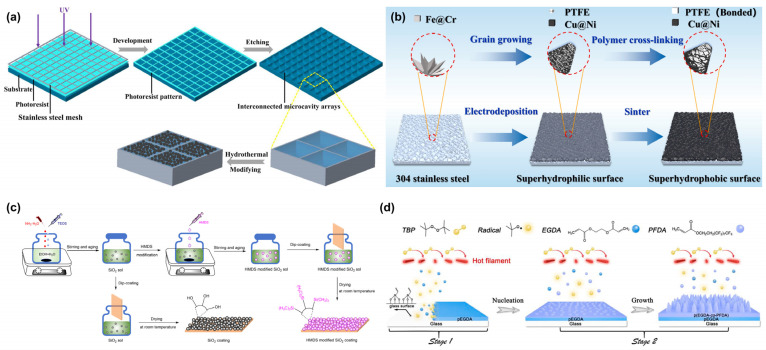
Preparation of superhydrophobic surfaces by chemical methods: (**a**) chemical etching [62]; (**b**) electrochemical deposition [64]; (**c**) sol-gel method [67]; (**d**) vapor deposition [69].

Chemical etching is an effective method for fabricating superhydrophobic surfaces through selective dissolution to construct micro/nanostructures, with its core principle relying on the anisotropic corrosion effect of acid/alkali solutions on material surfaces (Figure 5a) [62]. This approach involves immersing metal or silicon substrates in specific etchants (such as HCl/H_2_O_2_ mixtures or NaOH solutions), where surface roughness is generated in situ by precisely controlling corrosion time, concentration, and temperature. Subsequent low-surface-energy modification yields surfaces with high contact angles and low sliding angles. This technique offers the advantages of simple equipment and low cost, making it particularly suitable for large-area treatment of metallic materials like aluminum alloys. Wei et al. [63] cleaned Mg-9Al-1Zn (AZ91) alloy plates by immersion in a 0.3 mol/L HCl aqueous solution, followed by sequential cleaning with deionized water and ethanol. The etched AZ91 magnesium alloy was then treated by room-temperature immersion in a 0.01 mol/L stearic acid ethanol solution, with a final rinsing in anhydrous ethanol and drying under hot air flow. The resulting superhydrophobic AZ91 alloy exhibited a dense microstructure, demonstrating enhanced Cassie-state stability and superior corrosion resistance. The surface maintained excellent chemical stability across a wide pH range and showed remarkable thermal stability. This study provides important insights for designing corrosion-resistant superhydrophobic surfaces and surfaces with tunable adhesion.

#### 3.2.2. Electrochemical Deposition

Electrochemical deposition is a chemical synthesis method that enables in situ growth of micro/nano structures on conductive substrates through electrochemical regulation (Figure 5b) [64]. This technique utilizes metal salt solutions as electrolytes, where micro/nano hierarchical structures are constructed on electrode surfaces by precisely controlling current density, deposition time, and additives. Notable for its low energy consumption and excellent substrate conformability, this approach offers significant advantages for scalable fabrication.

A particularly innovative study by Wang et al. [65] demonstrated the fabrication of a highly stable and multifunctional superhydrophobic PTFE coating on stainless steel. Their method employed a preparation solution containing poly(tetrafluoroethylene) (PTFE), MgCl_2_, and water dissolved in anhydrous ethanol, followed by electrodeposition and high-temperature curing. This process allows for the rapid (minutes-scale) formation of superhydrophobic PTFE coatings on any metallic surface, with tunable surface morphology and hydrophobicity by adjusting the water content during electrodeposition. The resulting PTFE coating exhibits outstanding performance, including exceptional stability, self-cleaning properties, corrosion resistance, and anti-scaling capability. With its cost-effectiveness and engineering adaptability, this method holds significant promise for industrial applications, such as marine coatings, heat exchangers, and anti-icing systems.

#### 3.2.3. Sol-Gel Method

The sol-gel method is a chemical synthesis method in which a gel system is obtained by hydrolysis of precursors in the liquid phase and then colloidal particles through a condensation reaction (Figure 5c). The nanoparticles in the system are roughened on the surface of the substrate material by physical deposition or chemical adsorption [66]. A representative example is the work by Ye et al. [67], who developed a superhydrophobic SiO_2_ anti-reflective coating with both high optical transparency and exceptional environmental stability through a simple two-step sol-gel process. By synergistically modulating the coating’s refractive index using water and hexamethyldisilazane (HMDS), they achieved an optically optimized SiO_2_ coating with a tunable refractive index ranging from 1.18 to 1.23. This precisely engineered refractive index range enables perfect matching with various common substrates

#### 3.2.4. Vapor Deposition

Chemical vapor deposition (CVD) is a method wherein certain substances in solution react with a modified material to form a new substance through a gas phase reaction. This forms a micron- or nano-sized film with a certain roughness on the surface of the material, thus achieving the effect of modification [68]. For instance, Huang et al. [69] prepared transparent superhydrophobic polymer nanocone array coatings with excellent anti-icing properties through a simple one-step CVD method (Figure 5d). The novel CVD process employed condensed nanoscale monomer droplets as nucleation centers, followed by the growth of vertically aligned polymer nanocones. The height and density of the nanocones can be well controlled by adjusting the deposition conditions. The optimized nanocone array coating has excellent self-cleaning capability and mechanical durability against water droplet and grit impacts, and it has potential practical applications in outdoor environments.

### 3.3. Self-Assembly Method

#### 3.3.1. Molecular Self-Assembly

**Figure 6:**
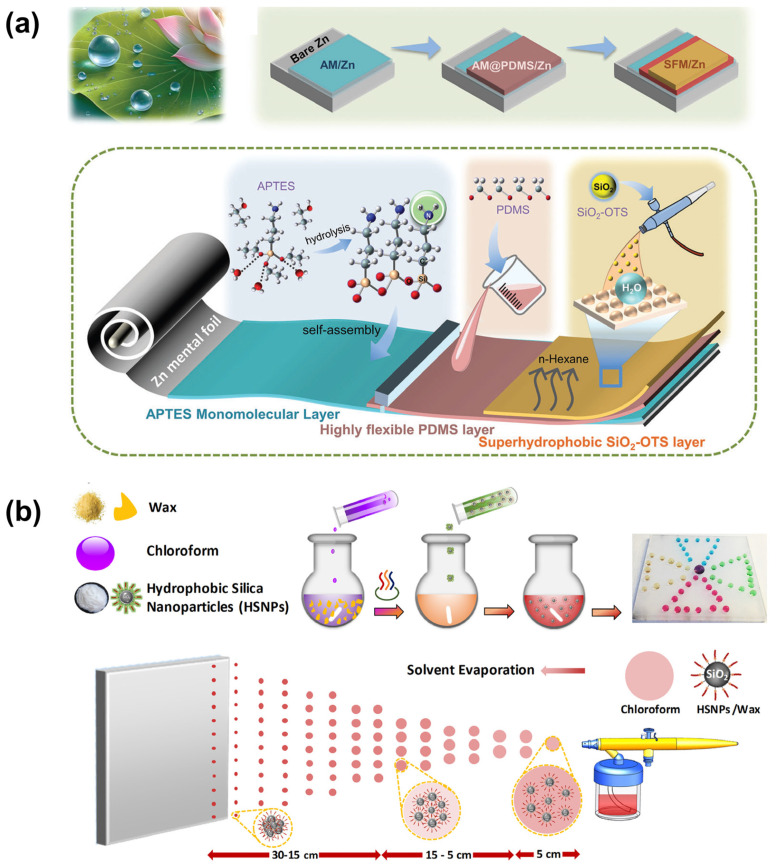
Preparation of superhydrophobic surfaces by self-assembly methods: (**a**) molecular self-assembly [71]; (**b**) nanoparticle self-assembly [73].

Molecular self-assembly is a surface modification technique that utilizes weak intermolecular interactions, such as van der Waals forces and hydrogen bonding, to spontaneously form ordered monolayers (Figure 6a) [70]. In this process, substrates are immersed in solutions containing thiol or silane compounds. The active terminal groups of these molecules chemically bond with the substrate surface, while the hydrophobic alkyl or fluorocarbon chains orient outward, resulting in highly ordered monolayers. This molecular-level assembly effectively reduces surface energy. When combined with pre-constructed micro/nanoscale rough structures, it yields superhydrophobic surfaces exhibiting high apparent contact angles. Due to the uniformity and controllability of these monolayers, this technique is particularly suitable for precision devices requiring atomic-level flatness. For example, integrating molecular self-assembly with spray-coating techniques has led to the development of superhydrophobic, highly flexible artificial solid–electrolyte interphase (SEI) layers on zinc (Zn) anodes. These layers effectively buffer the growth of Zn dendrites by mitigating the “tip effect”, thereby suppressing dendrite formation. Such advancements are crucial for the commercial application of Zn anodes in energy storage systems [71].

#### 3.3.2. Self-Assembly of Nanoparticles

Nanoparticle self-assembly is a strategy that constructs micro/nano hierarchical structures through the orderly stacking of nanomaterials. This approach leverages the self-organizing behavior of nanoparticles (such as SiO_2_ or TiO_2_) on substrate surfaces to form porous, rough structures (Figure 6b) [72]. Typically, monodisperse nanoparticle suspensions are deposited onto substrates using techniques like dip-coating, spin-coating, or spraying. Subsequent thermal treatment at 150–300 °C enhances interparticle bonding. The surface is then modified with fluorosilanes via vapor deposition to reduce surface energy. By adjusting particle size distribution and packing density, superhydrophobic coatings with high static contact angles and low sliding angles can be achieved. The inherent properties of nanoparticles also impart self-cleaning functionalities to the surface. This method offers advantages such as simplicity, low cost, and broad substrate compatibility. An intriguing development involves a one-step spray-coating technique that creates robust superhydrophobic surfaces. This method utilizes a dispersion composed of alkyl-silane-functionalized nanoparticles and wax [73]. By controlling solvent composition and spray distance, surfaces with a water contact angle of 175° and a sliding angle of 3° are achieved. The resulting coatings maintain their superhydrophobic properties even after 1000 cycles of water spray impact, 45 min of water jet exposure, and 180 cm of linear abrasion. This low-cost, scalable, fluorine-free fabrication process can be applied to virtually any material surface, offering significant potential for practical applications.

## 4. Applications

Biomimetic superhydrophobic surfaces, inspired by natural structures, have demonstrated significant application potential across various fields. In the realm of self-cleaning, architectural coatings that mimic the micro/nano structures of lotus leaves have been developed to extend cleaning intervals by reducing dust accumulation and facilitating water runoff. In aerospace, anti-icing coatings for aircraft have been engineered by emulating the directional microstructures found on water strider legs, effectively minimizing ice formation on critical surfaces. In the energy sector, dual-functional optical-hydrophobic coatings inspired by butterfly wings have been applied to solar panels, maintaining high light transmittance while providing self-cleaning capabilities, thereby enhancing energy conversion efficiency. These biomimetic superhydrophobic surfaces capitalize on the functional advantages of biological prototypes, addressing engineering challenges such as mechanical durability and scalability. Their development marks a transition from laboratory research to industrial applications, underscoring the transformative impact of nature-inspired designs in advanced material engineering.

### 4.1. Self-Cleaning

Superhydrophobic surfaces have demonstrated revolutionary potential in self-cleaning applications by emulating the “lotus effect”. This effect is achieved through the construction of micro- and nanoscale rough structures combined with low-surface-energy chemical modifications, resulting in exceptional water repellency. When water droplets contact such surfaces, they form nearly spherical shapes that effortlessly roll off, carrying away dust and contaminants. Typical applications include self-cleaning coatings for building exteriors, anti-dust films for solar panels, and anti-fouling treatments for automotive glass. These coatings not only reduce maintenance efforts but also enhance the longevity and performance of the underlying materials.

**Figure 7:**
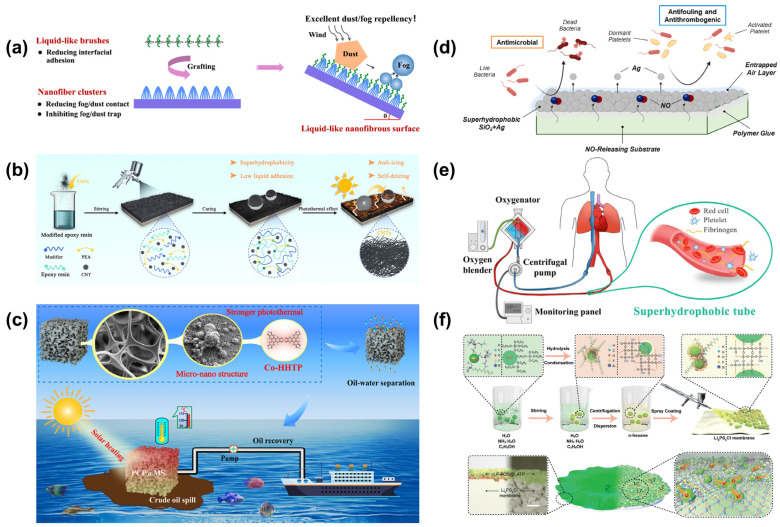
Application of superhydrophobic surfaces: (**a**) self-cleaning and anti-dusting [76]; (**b**) anti-icing [77]; (**c**) oil–water separation [80]; (**d**) antimicrobial [82]; (**e**) biomedicine tubes [81]; (**f**) solid electrolyte membrane [83].

An intriguing example is presented by Boutamart et al. [74], who developed a fluorine-free superhydrophobic coating for concrete substrates using a sol-gel method. This coating, composed of low-surface-energy PDMS and polymeric silica (PS), was applied via a dip-coating technique. It simultaneously modified the microstructure and chemical properties of the concrete surface, resulting in excellent superhydrophobic performance with a water contact angle exceeding 150°. The coated concrete substrates demonstrated resistance to dust contamination and withstood over 300 cycles of abrasion tests. Moreover, the coating exhibited good weather resistance under various conditions, including temperature fluctuations, ultraviolet radiation, and erosive wear. Wang et al. [75] developed a fluorinated superhydrophobic coating for photovoltaic modules by spraying a solution of SiO_2_ nanoparticles onto glass surfaces. This coating forms a micro/nanostructured anti-reflective layer that enhances light transmittance, thereby improving the power generation efficiency of photovoltaic modules under harsh environmental conditions. Additionally, the superhydrophobic film exhibits excellent self-cleaning properties, significantly reducing dust accumulation on the surface of the photovoltaic modules and further enhancing their efficiency. Another remarkable example is presented by Dou et al. (Figure 7a) [76], who employed a straightforward plasma-etching technique to fabricate surfaces featuring subwavelength nanofiber cluster structures. Subsequent modification with liquid-like perfluoropolyether resulted in a superhydrophobic surface exhibiting exceptional optical transparency. This surface achieved a static water contact angle of 171° and a sliding angle as low as 0.5°, demonstrating outstanding self-cleaning capabilities. The synergy between the nanofiber cluster architecture and the smooth, liquid-like surface chemistry effectively suppressed the accumulation of dust particles and fog droplets. Notably, the surface maintained excellent optical transparency even after 24 h of fog exposure, highlighting its potential for applications requiring both transparency and water repellency.

### 4.2. Anti-Icing/Anti-Fogging

Superhydrophobic surfaces offer significant advantages in anti-icing and anti-fogging applications by leveraging micro/nano composite structures and low surface energy to effectively delay ice formation and suppress fog accumulation. In the aerospace sector, applying superhydrophobic coatings to aircraft wings can extend the onset time of icing and reduce ice adhesion strength, enhancing flight safety and performance. In optical devices, anti-fog lenses inspired by butterfly-wing structures can maintain clarity by increasing fog-free duration. The core mechanism involves air layers trapped within micro/nanostructures, which lower the probability of ice nucleation, while surface chemical modifications enable condensed water droplets to roll off swiftly, preventing fog formation. These features make superhydrophobic surfaces highly effective for passive anti-icing and anti-fogging solutions across various industries. Yang et al. (Figure 7b) [77] developed a superhydrophobic photothermal coating by integrating carbon nanotubes (CNTs) with modified epoxy resin through a spray-coating technique. The resulting surface exhibited a static water contact angle of 159.5° and extremely low liquid adhesion, indicating excellent water repellency. The modified epoxy resin provided robust chemical and mechanical stability, effectively preserving the micro/nanostructured roughness formed by the CNTs. Consequently, the coating maintained its superhydrophobic properties even after exposure to mechanical abrasion, chemical corrosion, and ultraviolet irradiation. Thanks to the superior light absorption and thermal conductivity of CNTs, the coating demonstrated efficient photothermal conversion. Upon solar irradiation, the surface temperature increased rapidly, enabling self-deicing within 400 s. This performance highlights the coating’s potential for application in aircraft anti-icing systems, contributing to enhanced flight safety. An et al. [37] drew inspiration from the vivid structural coloration and superhydrophobic properties of butterfly wings to design a cellulose nanocrystal (CNC)-based hierarchical photonic film. This film features a chiral nematic structure that imparts structural color and a superhydrophobic layer composed of polymer microspheres, which imparts water-repellent properties. The superhydrophobic layer includes “stomate”-like pores that allow humid air to permeate, enabling the film to function as a colorimetric humidity sensor. Additionally, the film demonstrates excellent self-cleaning and anti-fouling capabilities. When one side of the CNC film is coated with the superhydrophobic layer, the resulting Janus film exhibits asymmetric expansion and bending behaviors, as well as responsive structural colors in humid ethanol. These CNC-based hierarchical photonic materials hold promising applications in photonic sensors for extreme environments and smart photonic actuators.

### 4.3. Oil–Water Separation

In the field of oil–water separation, superhydrophobic surfaces have achieved high-efficiency separation through selective wettability. The core principle involves constructing superhydrophobic and superoleophilic properties on micro/nanostructured surfaces. Such materials can selectively block water while allowing oil to pass through rapidly, making them effective for treating oily wastewater and recovering marine oil spills. Oil–water separation tests are conducted in accordance with ASTM and ISO standards. The separation efficiency was quantified using infrared spectroscopy to analyze the oil content of the aqueous phase before and after separation. Flux testing was performed by measuring the separation flux per unit time (L·m^−2^·h^−1^) at a pressure of 0.1 bar. Wang et al. [78] employed a thermally induced nonsolvent phase separation (TINIPS) method to fabricate superhydrophobic/superoleophilic reduced graphene oxide/polycarbonate (RGO/PC) porous monoliths. These monoliths exhibited a high specific surface area of 137.19 m^2^/g and a porosity of 91.3%. They demonstrated excellent superhydrophobicity (water contact angle of 161°) and superoleophilicity (oil contact angle of 0°), along with stable repellency against acidic and alkaline solutions. The prepared porous monoliths effectively separated various oils and organic solvents from water, exhibiting fast adsorption rates, high adsorption capacities, and good recyclability, making them important for oil spill and chemical leak cleanup.

Yin et al. [79] developed a multifunctional cellulose-based membrane with superhydrophobic properties and excellent functionalities, including self-cleaning, oil–water separation, antifouling, and photocatalytic degradation capabilities. The membrane was fabricated using a simple solution immersion method. The oil–water separation efficiency reached 96%, with high flux and recyclability, attributed to the synergistic effects of the CuO nanostructure coating and the stearic acid modification, resulting in strong superhydrophobicity. The membrane exhibited a water contact angle of approximately 160° and maintained high stability against exposure to acidic, alkaline, and saline solutions, as well as under flexible bending and UV irradiation. Additionally, the multifunctional cellulose-based membrane preferentially adsorbed organic dyes, such as methylene blue, from aqueous solutions, promoting their efficient degradation under UV irradiation and demonstrating high recyclability. Notably, due to the self-cleaning and antibacterial properties of CuO nanoparticles, the membrane exhibited excellent antifouling ability, resisting algae and bacterial adhesion for extended periods, making it suitable for wastewater treatment in complex environments.

Crude oil’s high viscosity and low fluidity make effective spill cleanup a significant challenge. To address this, He et al. (Figure 7c) [80] developed a composite sponge (PCP@MS) using a layer-by-layer self-assembly method. They embedded Co-HHTP photothermal materials and coated the surface with low-surface-energy PDMS on melamine sponge substrates. The resulting composite sponge exhibited excellent superhydrophobicity, antifouling properties, and chemical stability in harsh environments, including acidic, alkaline, and simulated seawater conditions. The sponge’s internal micro/nano hierarchical structure enhanced light absorption, and the Co-HHTP photothermal conversion properties enabled the PCP@MS to rapidly heat up under 1.0 solar irradiation within 300 s, effectively reducing crude oil viscosity. Under irradiation, it achieved an impressive absorption capacity of 62.5 g/g for ultra-high-viscosity crude oil. Additionally, the PCP@MS maintained its absorption capacity after 10 compression cycles, with a high recovery rate of 92%. Utilizing a peristaltic pump, the composite sponge demonstrated continuous oil absorption from seawater surfaces, achieving a delivery rate of 8.76 g/min. This in situ solar-assisted heating adsorbent design holds promise for advancing practical applications in viscous oil spill cleanup and recovery.

### 4.4. Biomedicine

Superhydrophobic surfaces have demonstrated significant potential in the biomedical field, primarily due to their exceptional resistance to biofouling and their ability to modulate interfaces. By creating micro- and nanostructured rough surfaces and modifying them with low-surface-energy fluorosilanes, these surfaces can substantially reduce the adhesion of proteins and bacteria. This has led to successful applications in the surface treatment of medical devices such as catheters and orthopedic implants, significantly lowering the incidence of postoperative infections. In the realm of drug delivery, superhydrophobic surfaces inspired by rose petal structures enable precise loading and release of minute drug quantities. Additionally, stimuli-responsive superhydrophobic materials, like pH-sensitive polymer coatings, facilitate the development of intelligent drug delivery systems. These advancements underscore the transformative impact of superhydrophobic surfaces in enhancing the performance and safety of medical devices and therapeutic systems.

An interesting study by Sun et al. (Figure 7e) [81] developed superhydrophobic blood-repellent tubes to replace conventional heparin-coated tubes in clinical ECMO (extracorporeal membrane oxygenation) treatments. These tubes demonstrated excellent superhydrophobicity and blood-repelling properties, leading to prolonged coagulation times and reduced protein and platelet adhesion. Specifically, the superhydrophobic-treated tubes clotted in 36 min, compared to 21 min for clinical Bioline heparin-coated tubes. Additionally, protein and platelet adsorption on the superhydrophobic-treated tubes decreased by 32% and 74%, respectively. In vivo and in vitro biotoxicity tests further confirmed that the superhydrophobic-treated tubes were non-toxic.

Ozkan et al. (Figure 7d) [82] combined bioinspired superhydrophobicity with nitric oxide (NO) release to develop a novel anti-wetting biomaterial aimed at mitigating bacterial infections and thrombosis associated with blood-contacting medical devices. They fabricated superhydrophobic surfaces by dip-coating medical-grade silicone rubber with hydrophobic SiO_2_ and silver nanoparticles, creating a hierarchical micro/nanostructure with low surface energy. The incorporation of S-nitroso-N-acetylpenicillamine (SNAP), an NO donor, endowed the material with antimicrobial and antithrombotic properties. This multifunctional surface significantly reduced adhesion of Escherichia coli, Staphylococcus aureus, and platelets, while exhibiting no cytotoxic effects on fibroblast cells

### 4.5. Energy and Electronics

Superhydrophobic surfaces have demonstrated significant potential in the energy and electronics sectors, owing to their exceptional water-repellent, anti-fouling, and interfacial control properties. In the energy field, incorporating superhydrophobic separators in lithium–sulfur batteries effectively suppresses the polysulfide shuttle effect, thereby extending the battery’s cycle life. For photovoltaic panels, applying self-cleaning superhydrophobic coatings reduces dust accumulation, enhancing light transmittance and improving photoelectric conversion efficiency. In electronics, superhydrophobic encapsulation materials help maintain the insulation performance of circuit boards in high-humidity environments.

Xu et al. (Figure 7f) [83] reported the first water-stable sulfide solid electrolyte membrane, achieved by applying a superhydrophobic, lithium-ion-conductive coating. This protective layer not only prevents moisture intrusion but also facilitates efficient Li^+^ transport within all-solid-state batteries. The coating was created by spray-coating Li_1·4_Al_0·4_Ti_1·6_(PO_4_)_3_ (LATP) nanoparticles encapsulated with fluorinated polysiloxane (F-POS), synthesized through the hydrolysis and condensation of tetraethyl orthosilicate (TEOS) and 1H,1H,2H,2H-perfluorodecyltriethoxysilane (PFDTES) molecules. The LATP nanoparticles provide Li⁺ transport pathways and microscale roughness, while the F-POS imparts nanoscale roughness and low surface energy, resulting in a superhydrophobic surface with a water contact angle exceeding 160°. This modified sulfide membrane can withstand extreme exposure conditions, such as 70% relative humidity and direct water jetting, without compromising its electrochemical performance. The method offers a simple and effective post-treatment applicable to various sulfide and other air/moisture-sensitive materials.

Ding et al. [84] reported a simple and scalable method to fabricate stretchable superhydrophobic surfaces with tunable surface microstructures, including arc-shaped and V-shaped air pockets, for multi-stage droplet microreactors. Surfaces with arc-shaped air pockets maintain a stable Cassie–Baxter state, enabling droplet-based microreactions. In contrast, surfaces with V-shaped air pockets facilitate pressure-induced Cassie–Baxter to Wenzel transitions, promoting droplet transport between reaction sites. This low-cost and scalable fabrication approach offers new strategies for potential applications of digital microfluidics in stretchable devices for biomedical analysis and biochemical sensing.

For the application of superhydrophobic surfaces in the above-mentioned fields, researchers have used different approaches to realize their specific applications, which are briefly summarized in Table 1:

**Table 1:** Comparative summary of preparation methods of superhydrophobic surfaces for different applications.

Application	Preparation Method	Advantage	Reference
Self-cleaning	Sol-gel, Template method	Low cost, suitable for large-scale preparation, multi-functional integration	[85]
			[86]
Anti-icing/ Anti-fogging	Laser etching, CVD	Metal substrate, flexible substrate, high-precision manufacturing of the structure	[87]
			[88]
Oil–water separation	Chemical etching, self-assembly of nanoparticles	Easy to scale up, large-area preparation, wide range of substrate adaptability	[89]
			[90]
Biomedicine	Plasma processing, layer by layer self-assembly	Precise regulation of surface chemistry, molecular-level thickness control	[91]
			[92]
Energy and electronics	Atomic layer deposition,	High-throughput, atomic precision	[93]
	spray method		[94]

Currently, the industrial application of superhydrophobic surfaces has gradually moved from the laboratory to actual production, but it still faces the challenges of cost, durability, and large-scale production. Therefore, the industrialization of superhydrophobic technology should select the most cost-effective manufacturing method according to the application scenarios, while continuously optimizing the durability and compatibility with large-scale production. In the future, smart responsive materials, green processes, and self-healing technologies will be the breakthrough direction for superhydrophobic surfaces.

## 5. Conclusions

Research into biomimetic superhydrophobic surfaces has unveiled the remarkable ingenuity of natural organisms—such as lotus leaves, water striders, and butterfly wings—in designing multiscale structures and regulating surface functions. These insights have inspired innovative paradigms for developing artificial functional surfaces. Currently, by integrating micro/nano hierarchical structures with low surface energy modifications, superhydrophobic technologies have achieved groundbreaking applications in the self-cleaning, anti-icing and anti-fogging, oil–water separation, and biomedical fields. For instance, biomimetic coatings have enhanced solar panel efficiency by 10%, and antibacterial catheters have reduced infection rates by over 90%. However, large-scale applications still face challenges such as limited durability, environmental concerns, and adaptability to complex conditions. There are several areas that future research should focus on (1) In terms of water scarcity, designing efficient water-collecting surfaces to collect atmospheric moisture in arid regions to alleviate water scarcity; (2) In terms of improving energy efficiency, developing self-cleaning photovoltaic coatings to improve the efficiency of solar panels and help clean energy transition; (3) In terms of environmental protection, replacing traditional fluorine-containing coatings with non-fluorinated silicone to avoid the accumulation of toxicity in living organisms.

Furthermore, interdisciplinary integration—such as with flexible electronics and biomimetic robotics—will foster emerging applications of superhydrophobic technologies in deep-sea exploration, space protection, and wearable devices. Over the next decade, breakthroughs in green manufacturing and intelligent materials are expected to position biomimetic superhydrophobic surfaces as a key technology for addressing challenges in the energy, environment, and healthcare fields while promoting sustainable development that harmonizes with nature.

## Data Availability

No new data were created or analyzed in this study. Data sharing is not applicable to this article.
